# Time to Call into Question the Fundus-based Evaluation of Diabetic Retinopathy after Intravitreal Injections

**DOI:** 10.18502/jovr.v15i1.5971

**Published:** 2020-02-02

**Authors:** Ramin Tadayoni

**Affiliations:** ^1^Université de Paris, Ophthalmology Department, AP-HP, Hôpital Lariboisière, F-75010, Paris, France

Diabetic retinopathy (DR) scores have two fundamental objectives: (1) detecting DR complications and their severity and (2) predicting the risk of progression to DR complications when they are not yet present. Detecting complications is the easiest part as it only requires to properly evaluate the existing situation such as finding new vessels or an edema. On the contrary, predicting the risk of progression is the most challenging part as it involves predicting the future.

Over the last century, the Airlie house classification group and later the Early Treatment Diabetic Retinopathy Study Research (ETDRS) group have done an outstanding work, even with the current standard, to create and improve a scoring to be used to predict the risk of DR progression before the occurrence of complications.^[1]^ Not all diseases and complications can be predicted with the exploration tools available. For example, the occurrence of diabetic macular edema cannot yet be appropriately predicted. Conversely, proliferative DR might be predicted based on the signs seen on fundus examination or, even better, on fundus photos.^[1]^ The ETDRS group has done a very precise step-by-step work, evaluating not only the value of the signs but also the reproducibility of their evaluation, to identify the most suitable signs. Their choices were also based on pragmatism. They did not have all the currently available imaging modalities, so they have mainly used 7-field fundus photos covering for that time a satisfactory wide surface of the fundus. Eventually, fluorescein angiography has been found to be slightly more powerful than standard photos. However, as it is an invasive technique, the extra power supplied has not been considered justified given the treatments available at that time.^[2]^ The diabetic retinopathy severity scale (DRSS) and its simplified versions have finally become the gold standard for evaluating DR in clinical studies and for treating patients.

DR pathophysiology is better known today and it is obvious that, before the occurrence of proliferation, fundus signs act as surrogates for diabetes-related changes: deep hemorrhages are signs of capillary non-perfusion that leads to ischemia and vascular endothelial growth factor (VEGF) secretion, while venous beading is a feature of vessel impregnation with VEGF for example. Some hemorrhages may eventually disappear over time despite persistent non-perfusion but if the non-perfusion area expands, new hemorrhages appear. Therefore, the higher the extand of hemorrhages is, the more new non-perfusion areas occur, and the higher the risk of progression to proliferation is. It should be noted that these signs do not correspond to the disease itself (*i.e.* retinal héamorrahges per se are not the problem), but are used to estimate the risk of DR progression to its complicated forms. The DRSS and its variants have been used for so long that over time many clinicians have ended up merging these surrogate signs with their meaning and the disease itself. Thus, a reduction in hemorrhages has become the equivalent of DR improvement. This might have been acceptable as far as there was no mean to dissociate disease progression and its surrogate signs.

Intravitreal injections, in particular anti-VEGF agents, have since been developed and used for the treatment of diabetic macular edema. As expected from an effective anti-angiogenic agent, anti-VEGF drugs have been shown to be able to control new vessels in DR eyes. More interestingly, it has also been shown that the DRSS score based on color fundus photos could improve after anti-VEGF intravitreal injections.^[3,4]^ For these reasons, they have immediately been labeled as agents improving DR. In case of regression of a complication of DR, including new vessels or edema, it may be legitimate to call it DR improvement. However, in case of reduction in signs of non-complicated DR, this can only be acceptable if the risk of progression is proportionally reduced. In other words, if, after anti-VEGF injection, the ETDRS-DRSS fundus photo score decreases from 53, that is, severe non-proliferative diabetic retinopathy (NPDR), to 35, that is, mild NPDR, the risk of progression to proliferative DR during the year should decrease from > 50% to < 10%, and it should evolve at least as any mild NPDR that is likely to progress to severe NPDR usually several years thereafter.

Data on DR evolution after anti-VEGF treatment are limited but the reported series tend to suggest that anti-VEGF intravitreal injections clear the fundus from hemorrhages and signs of vessel impregnation with VEGF without eliminating the risks of neovascularization shortly after treatment discontinuation.^[5]^ This may indicate that despite a DRSS score improvement, ischemia persists. To explore this assumption, we have conducted two successive studies evaluating retinal perfusion after three anti-VEGF injections. In the first study based on fluorescein angiography, no vessel reperfusion was found despite an improved DRSS score on color photos. Indeed, even after this short treatment, new vessels, when present, regressed partly or totally. Fundus signs also improved in others. Then, the DRSS score improved by at least one stage in 61% of eyes. Meanwhile, in our study based on ultrawide-field fluorescein angiography, no significant reperfusion of arterioles or venules was observed in or around the non-perfusion areas.^[6]^ In the second study, using a similar method but based on wide-field OCT angiography, we found that despite a rapid DRSS score improvement after anti-VEGF treatment, no reperfusion occurred, including at the capillary level.^[7]^ Thus, our two studies have shown that the DRSS score can improve in the absence of reperfusion.

It is now the time to question the fundus-based evaluation of DR after intravitreal injections. When after intravitreal injection a severe NPDR (score 53 ETDRS-DRSS) changes its appearance to the one of a mild NPDR (score 35) on fundus photography but continue to have the non-perfusion of a score 53, will it evolve as a mild NPDR or as a severe NPDR? If one considers that the non-perfusion is the cause of ischemia and VEGF production leading to proliferation, unless other mechanisms are involved, the evolution should be closer to the evolution of a severe NPDR. This substantial doubt on the value of fundus-based ETDRS-DRSS scores invalidates relying only on color fundus for grading NPDR after intravitreal injections.

Waiting more data or a new method for assessing the risk of progression available, what may be the practical effects of such an uncertainty on the post-injection value of the ETDRS-DRSS scores? In clinical practice, if the injections are continued with short enough intervals, they may prevent any complications, but if they are discontinued, to be on the safe side, regardless of the fundus appearance, the risk should be considered the highest measured during the medical history of the eye and the follow-up should be decided accordingly. In some cases, OCT angiography or fluorescein angiography may also help to reevaluate the status of retinal perfusion. In upcoming clinical trials on this topic, it would be safe to include as much as possible multimodal imaging to compile data and also to be able to provide information required by the updated standards when they will end. We can indeed hope that in the forthcoming years, we will better understand with which modality and how we should evaluate DR for eyes treated by intravitreal injections. More generally, regardless of injections, isn't it time to try to switch from the historical classification of DR to new modalities using the best available modern images and all available data?
